# Association between anxiety disorder in young women and undifferentiated connective tissue dysplasia: circadian autonomic dysregulation and the “autonomic paradox”

**DOI:** 10.1192/j.eurpsy.2026.10649

**Published:** 2026-07-31

**Authors:** M. S. Artemieva, A. Shadrikova, N. Danilina, V. Sokolov, A. Lazukova, I. Kurnikova, A. Pavlov, S. Gulova, M. H. Khazaea Ali

**Affiliations:** Peoples’ Friendship University of Russia named after Patrice Lumumba, Moscow, Russian Federation

## Abstract

**Introduction:**

Undifferentiated connective tissue dysplasia (uCTD) in young women is frequently accompanied by autonomic dysregulation, heightened interoceptive sensitivity, and allostatic load—factors implicated in anxiety. The mechanisms linking uCTD severity with anxiety, particularly circadian autonomic signatures, remain under-characterised.

**Objectives:**

To test the association between uCTD severity and anxiety, and to examine whether circadian autonomic dysregulation (including the “autonomic paradox”) mediates this link.

**Methods:**

Cross-sectional study of 47 women (21–28 years) with verified uCTD. Two severity groups by dysplasia score: subclinical (n=24; DCT<23) vs manifest (n=23; DCT≥24). Anxiety was assessed with the Taylor Manifest Anxiety Scale (TMAS; 0–50). Autonomic proxies comprised 24-h heart-rate-variability (HRV) analysis with Kerdo Index (VIK) and spectral metrics (LF, HF, VLF, LF/HF) plus Index of Centralization (IC); day–night segmentation. Group comparisons and day/night correlations were analysed.

**Results:**

Anxiety was higher with greater uCTD severity (TMAS medians: 14 vs 25; difference=9). A tonic VIK shift occurred from marked vagotonia to formal sympathicotonia (−14.5±4.8 → +2.1±7.4; Δ=16.6; 95%CI 3.99–29.11; p<0.01). Despite sympathicotonia, LF/HF decreased by 23% (0.55±0.20 → 0.42±0.19) with LF −27% (1504→1093 ms^2^) and HF +17% (3329→3889 ms^2^)—constituting an “autonomic paradox.” VIK–HRV coupling strengthened at night (LF/HF r: 0.40→0.50; IC r: 0.45→0.55; VLF/HF r: 0.38→0.48). IC indicated pathological nocturnal centralization (physiologically expected to decline at night).

**Conclusions:**

Greater uCTD severity is associated with higher anxiety in young women and with circadian autonomic dysregulation characterised by an “autonomic paradox” and nocturnally reinforced VIK–HRV coupling. These objective metrics (TMAS, VIK/HRV, IC) may support psychiatric risk stratification, staging, and treatment monitoring. Clinically, subclinical uCTD may benefit from vagal-tone maintenance, whereas manifest uCTD warrants restoration of autonomic reciprocity and reduction of allostatic load (sleep regularity, graded activity, breathing-based arousal control, CBT/schema-therapy; pharmacotherapy as indicated).

**Disclosure of Interest:**

None Declared

